# Engaging Youth in a Co‐Design Process for Development of a Young Dementia Supporter Toolkit

**DOI:** 10.1002/alz70858_103317

**Published:** 2025-12-26

**Authors:** Kathy Hickman, Laura E. Middleton, Phyllis Fehr, William Heibein

**Affiliations:** ^1^ Alzheimer Society of Ontario, Toronto, ON, Canada; ^2^ Schlegel‐UW Research Institute for Aging, Waterloo, ON, Canada; ^3^ University of Waterloo, Waterloo, ON, Canada; ^4^ Dementia Empowerment Network, Hamilton, ON, Canada

## Abstract

Dementia‐friendly communities are places where people living with dementia and their care partners are understood, respected and supported. It is an environment where they feel more confident and able to participate in all aspects of community life because community members are educated about dementia and know how to engage with and support someone living with the condition. This begins with our youth. By creating supportive and inclusive communities of young people who understand the experience of dementia and the impact of ageism and stereotypes, we can change attitudes before stigma begins and have a long‐term impact on mental and social health and wellness of older adults living with dementia.

The Alzheimer Society of Ontario (ASO) in partnership with the Murray Alzheimer Research and Education Program (MAREP) at the Schlegel‐UW Research Institute for Aging (RIA) brought together a co‐design team of youth aged 9 to 16, people living with dementia, care partners, teachers, Alzheimer Society staff and researchers to develop a Young Dementia Supporter Toolkit. The toolkit was designed over xx virtual Zoom meetings and one full‐day in‐person workshop. The end product was a toolkit for youth, aged 9‐12 that equips young people with an understanding of dementia and practical strategies to tackle stigma and support people living with dementia in their families, local communities and beyond by becoming dementia supporters.

This presentation will share the co‐design process, the contents of the Young Dementia Supporter Toolkit, pilot test feedback results from youth volunteers, and evaluation of the development process including the experiences of co‐design team members. Project leads and co‐design team members will present the findings.

